# A Vibration Signal-Based Active Noise Control Method for Liquid-Filled Pipelines

**DOI:** 10.3390/s25020463

**Published:** 2025-01-15

**Authors:** Yunhao Wang, Qichao Liu, Wenjing Yu, Guo Cheng

**Affiliations:** 1 Laboratory of Vibration and Noise, Naval University of Engineering, Wuhan 430033, China; z24182405@nue.edu.cn (Y.W.);; 2 National Key Laboratory of Vibration and Noise on Ship, Naval University of Engineering, Wuhan 430033, China

**Keywords:** fluid–solid coupling, liquid-filled pipeline, active noise control, low-frequency line spectrum

## Abstract

Pulsation noise in the piping system generated by the excitation of the pump source seriously affects the reliability of the pipeline system and mechanical equipment. The active noise control can effectively suppress the low-frequency noise in the liquid-filled pipeline. Active control methods with intrusive secondary sources generally use dynamic pressure sensors or hydrophones to collect signals, which destroy the structure of the pipe. In this paper, we propose an active noise control method utilizing signals acquired by accelerometers, which adopts offline modeling of the secondary path and the notch narrowband FxLMS algorithm for controlling the secondary source actuation. The feasibility of this method is verified by LabVIEW simulation and active noise control test of the liquid-filled pipeline. The test results show that this method can achieve more than 4 dB reduction for low-frequency (10~200 Hz) line spectrum noise under most operating conditions.

## 1. Introduction

With the development of industrial technology, liquid-filled pipelines play an increasingly important role in aerospace, marine industry, petrochemical, and other fields. When the liquid-filled pipeline is working, the mechanical equipment connected to the pipeline will transmit noise to the outside through the vibration of the connecting parts and the liquid pressure pulsation in the pipe [1,2,3]. The vibration and noise of the liquid-filled pipeline will reduce the reliability of the equipment and affect the comfort of the working environment. Mechanical devices such as pumps and valves also radiate noise to seawater through the seaward piping, causing noise pollution [4,5].

At present, with the widespread use of vibration-damping elements such as elastic bases, mechanical vibration transmitted by the support components along the pipeline can be suppressed. Flow noise and flow-induced vibration caused by periodic pressurization of pumps have become the main source of noise in liquid-filled pipelines [6]. In liquid-filled piping systems, noise suppression is generally performed by installing flexible hoses, dampers, and mufflers [7]. In [8,9], Hou et al. proposed an elastic back cavity microperforated pipe anechoic structure. They applied the microperforated warm sound structure to the field of water pipeline noise control. It was combined with the elastic pipe wall to form an elastic back cavity, which broadened the bandwidth of anechoic sound elimination. Yan et al. [10] proposed an elastic liquid-filled muffler with a novel elastic back cavity and airbag structure, which achieved resonance muffling by generating pressure fluctuation in the opposite phase to the input flow through the forced vibration of the elastic plate. The test verified that the device achieved a sound muffling effect of 9.6 dB at the pipe mouth.

Passive control methods can effectively suppress vibration and noise in middle- and high-frequency bands. However, under high pressure, the vibration isolation effect of flexible hoses will deteriorate or even fail. Passive shock absorbers and mufflers also have disadvantages such as narrow control bands, limited application scenarios, and bulky size [11,12]. None of the above methods are ideal for controlling low-frequency vibration and noise [13]. As the main source of vibration and noise in the liquid-filled pipeline, the vibration and noise generated by the pump are mainly concentrated in the middle- and low-frequency bands [4]. The active control method has a significant suppression effect on low-frequency vibration and noise, and its application to liquid-filled pipelines has been a research hotspot [12]. Zhang et al. [14] used macro-fiber composites (MFCs) for active vibration control of fluid conveying pipelines and analyzed the effect of controller parameters on the effectiveness of active control. Pan et al. [15] proposed an integrated method for fluid noise suppression. They provided a composite control of fluid noise in hydraulic pipelines by combining an active feedforward noise attenuator with a flexible hose, which has the control effect of both high-frequency and low-frequency noise. Sun et al. [16,17] designed a pump water circulation pipe system using a pressure-balanced electromagnetically driven sound source to achieve active noise control. The system uses a line structure cascaded adaptive notch filter to generate control signals, which has good frequency tracking performance and single-frequency noise reduction. Buker et al. [18] developed an intrusive fluid power actuator, which controls the movement of the piston in the pipeline through a piezoelectric driver and emits a plane wave field to counteract pulsating waves in the pipe. Buker et al. used this device to carry out an experimental study on a centrifugal pump and significantly reduced the pulsating pressure in the downstream pipeline. Many studies have shown that the noise and vibration of liquid-filled pipelines are closely related to the pulsation of liquid pressure in the pipes [19,20]. However, existing studies on active noise control for liquid-filled pipelines use dynamic pressure sensors or hydrophones to obtain signals. This method requires making holes in the pipe wall and burying the sensors, which will damage the structure and reduce the strength and reliability of the pipes. Therefore, there is a need to carry out research on active noise control of liquid-filled pipelines from the perspective of improving signal acquisition methods.

In this paper, a vibration signal-based active noise control method for liquid-filled pipelines is proposed. In this method, the vibration signal is collected by accelerometers on the pipe wall. A notch narrowband FxLMS algorithm is used to process the signals and control the actuation of the secondary source. Firstly, the fluid–solid coupling between liquid pressure pulsation and pipe wall vibration is analyzed according to the working characteristics of the liquid-filled pipeline. Then, an active noise control system based on adaptive notch filters is designed, and the feasibility is proved by LabVIEW simulation. Finally, an active noise control test is carried out by setting up a pipeline circulation system. The test results show that the method can realize effective control of low-frequency line spectrum noise.

## 2. Basic Principles and Methods

### 2.1. Fluid–Solid Coupling Mathematical Model of Liquid-Filled Pipeline

When the pump source operates, it generates periodic flow fluctuations in the pipeline, which is called pressure pulsation. As the pressure pulsation propagates in the pipeline, it will not only propagate downstream in the form of flow noise but also stimulate vibration transmitted along the pipe wall through fluid–solid coupling. By solving the vibration response of the pipe wall under pressure pulsation excitation, the mathematical relationship between the two can be obtained. Based on the characteristics of piston pumps that periodically output flow, the expression of the pressure pulsation at the inlet pipe is given as follows:

(1) $$P_{0}=P_{ssc}+\sum_{i=1}^{N}P_{i}\sin(2\pi f_{i}t+\varphi_{i})$$

where $P_{i}$, $f_{i}$, and $\varphi_{i}$ represent the amplitude, frequency, and initial phase of the ith harmonic of the pressure pulsation, respectively. $P_{ssc}$ represents the steady state component of the liquid pressure pulsation. The harmonic frequency of the pressure pulsation can be calculated by

(2) $$f_{i}=\frac{nzi}{60}$$

where z denotes the number of plungers for the piston pump and n denotes the speed of the motor. To study the fluid–solid coupling vibration response of pipe walls under pressure pulsation excitation, the dynamic equation of liquid-filled pipeline is established according to the working condition and structural characteristics of liquid-filled pipeline system. The equation is based on the Bernoulli–Euler beam model and solved by the coupling characteristic line-finite element method [21]. The vibration response of the pipeline at time t obtained by solving is as follows:

(3) $$v_{0}=\sum_{i=1}^{N}A_{i}B_{i}P_{i}\sin(2\pi f_{i}t+\theta_{i})$$

where $\theta_{i}$ is the initial phase of the ith harmonic, $A_{i}$ is a coefficient characterizing the strength of the ith harmonic coupling and related to the modal intrinsic frequency of the pipeline, and $B_{i}$ is a coefficient related to the pipeline structure and coupling position.

By solving the fluid–solid coupling vibration response, it can be learned that the harmonic frequency of the vibration response is the same as that of the pressure pulsation. The vibration response under the excitation of pressure pulsation of different frequencies is mainly affected by the material parameters, boundary conditions, and pipeline structure. This means that the vibration of the pipeline is excited by pressure pulsations of the same frequency and the magnitude of the vibration amplitude is related to the intrinsic frequency of each order of modes.

### 2.2. Active Noise Control Method

**Figure 1:**
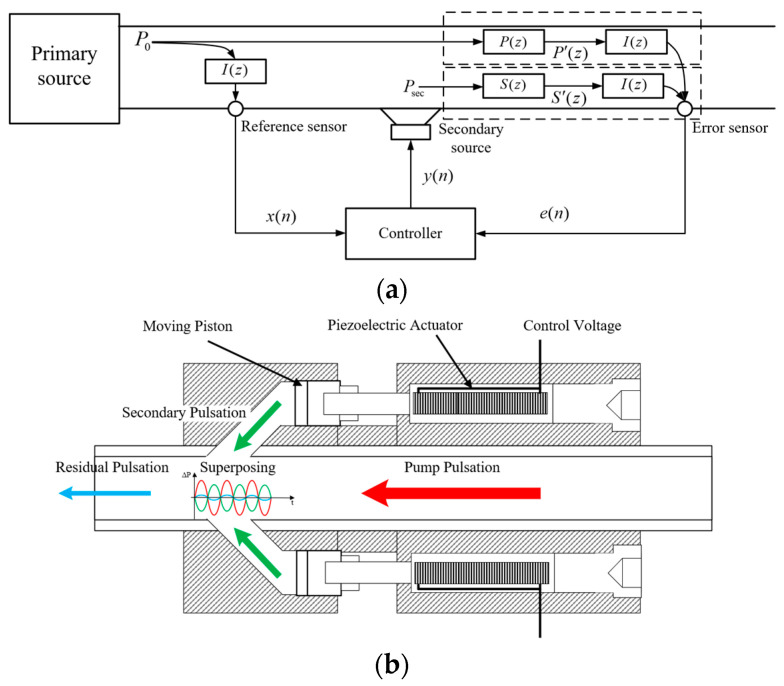
Principle of active noise control system. (**a**) Schematic diagram of the active noise control transmission paths; (**b**) principle of secondary source.

In active control methods for flow noise in liquid-filled pipelines, a secondary source is typically utilized to create a local volume change within the pipe, which in turn creates a secondary pulsation that propagates along the pipeline. The secondary pulsations are opposite in phase to the primary pulsations generated by the pump source and are superimposed to achieve attenuation of the pulsation noise amplitude. Once the vibration response of the pipeline under pulsating pressure excitation has been obtained, accelerometers can be used to collect the vibration signals on the pipe wall and thus characterize the pulsating pressure signals in the pipe. In this paper, a vibration signal-based active noise control method for liquid-filled pipelines is proposed, the basic principle of which is shown in Figure 1. In Figure 1a, P(z) and S(z) represent the primary and secondary paths for pressure pulsation transmission, respectively. For P(z), it represents an acoustic propagation path from the reference sensor to the error sensor. For S(z), it contains the controller peripheral circuitry and the acoustic propagation path from the secondary source to the error sensor. In this paper, I(z) is introduced to represent the coupling path between pressure pulsation and pipe wall vibration. The primary and secondary paths after the introduction of the coupling path are denoted as $P^{\prime }(z)$ and $S^{\prime }(z)$, respectively.

(4) $$P^{\prime }(z)=P(z)I(z)$$

(5) $$S^{\prime }(z)=S(z)I(z)$$

When active noise control is operating, primary and secondary pulsations pass through $P^{\prime }(z)$ and $S^{\prime }(z)$, respectively, and are attenuated at the error sensor. The remaining pulsation signal passes through the coupling path I(z) and is returned to the controller as an error signal e(n). Equations (4) and (5) give the expressions for the primary and secondary paths after adding the coupling path.

In this paper, an intrusive secondary source with side-branch structures is used, the principle of which is shown in Figure 1b. The secondary source is controlled by a controller outputting electrical signals of specified frequency, phase, and amplitude. Driven by four sets of piezoelectric actuators arranged along the pipeline, the pistons then generated actuation signals independently. The movement of the pistons produces a periodic volume change in the side-branch structures, which will propagate into the pipeline as a secondary pulsation that interferes with the pump pulsation. The secondary pulsations generated by this secondary source during operation can be expressed as

(6) $$P_{\sec}=\frac{V_{dn}S_{\sec}}{V_{ttl}S_{pip}}\rho_{f}c\lambda \sin(\omega_{s}t+\varphi_{s})$$

where $V_{ttl}$ and $V_{dn}$ represent the volume changes generated by the actuator in the side-branch structures and downstream of the pipeline, $S_{\sec}$ and $S_{pip}$ represent the piston cross-sectional area and the pipeline cross-sectional area, and $\rho_{f}$ and c represent the density and wave velocity of the liquid, respectively. The actuation displacement λ, frequency $\omega_{s}$, and phase angle $\varphi_{s}$ are controlled by the controller in real time.

The fluid–solid coupling vibration response of the pipeline under the action of secondary pulsation is solved according to the method described in Section 2.1, and the expression for the vibration response of the pipeline is obtained as

(7) $$v_{\sec}=ABC\lambda \sin(\omega_{s}t+\theta_{s})$$

In Equation (7), *A*, *B*, and *C* are used as coefficients to be determined concerning the intrinsic frequency of the pipeline, the solid and liquid parameters, and the structural elements of the system. Their influence on the control effect is contained in the secondary path $S^{\prime }(z)$, which can be determined by secondary path modeling. The principles of secondary path modeling are explained in Section 2.3.

### 2.3. Design of Adaptive Filter

The energy of the primary pulsations in the pipeline is mainly concentrated in the low-frequency line spectrum, consisting of the fundamental and harmonic frequencies, with a distinct narrowband characteristic. It has been learned in Section 2.1 that the pipeline vibration response is at the same frequency as the pulsating pressure excitation. Therefore, in this paper, active noise control is performed by adaptive notch filters based on the narrowband FxLMS algorithm. The algorithm can avoid the effects of secondary acoustic feedback since the reference signal is generated inside the program. Once the vibration acceleration signal obtained from the reference sensor is input to the controller, the peak line spectral frequency of the signal is extracted by the controller program, and the corresponding sinusoidal signal is generated as the reference input for the notch narrowband FxLMS algorithm.

**Figure 2:**
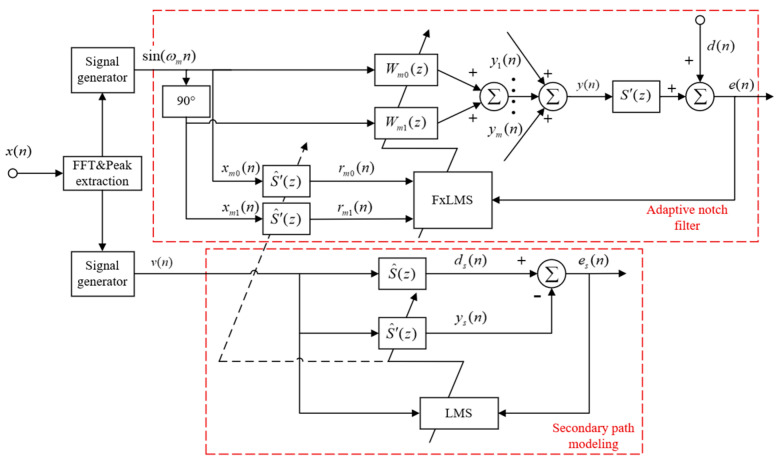
Block diagram of the notch narrowband FxLMS algorithm.

The structure of the notch narrowband FxLMS algorithm is shown in Figure 2. The active noise control section consists of m notch filters connected in parallel to control multiple noise line spectra [22]. Each notch filter consists of two weight coefficients, and the corresponding reference input signals are two program-generated sinusoidal signals with a 90° phase difference. The reference input signal is used for the iterative update of the notch filter weight coefficients after the secondary path estimation ${\hat{S}}^{\prime }(z)$. The update of the filter weight coefficients is given by Equation (8) and the output of the notch filter is given by Equation (9).

(8) $$w_{mi}(n+1)=w_{mi}(n)-2\mu e(n)r_{mi}(n),\;i=0,1$$

(9) $$y_{m}(n)=w_{m0}(n)x_{m0}(n)+w_{m1}(n)x_{m1}(n)$$

(10) M≈μtr[R]

The upper limit of the update step size μ usually depends on the maximum eigenvalue of the autocorrelation matrix R of the input signals. To balance the requirements of convergence speed and stability, however, it should be determined based on the statistical properties of the reference signal in the application scenario. Equation (10) presents a general rule for selecting the step size, where M is the amount of misalignment and tr[R] is the trace of R. For this paper, M is taken to be 10%, which is usually sufficient for stability. tr[R] can be obtained by measuring the power of the reference signal. The solution 0.0001 of μ obtained by rounding is used as the value of the update step size for both the simulation and test.

(11) $${\hat{s}}^{\prime }(n+1)={\hat{s}}^{\prime }(n)+2\mu_{s}x_{wn}(n)e_{s}(n)$$

In this paper, an offline modeling approach is used to model the coupled secondary path. Before active noise control begins, the peak line spectral frequency of the primary pulsation is first acquired. Then, the secondary source actuation is controlled by a program-generated narrowband white noise signal v(n). The signal received on the error sensor is used as the desired signal $d_{s}(n)$ for modeling, which differs from the output value $y_{s}(n)$ of the filter, and the resulting modeling error $e_{s}(n)$ is returned to the LMS algorithm. The LMS algorithm updates the weight coefficients of ${\hat{S}}^{\prime }(z)$ according to Equation (11). According to the principle mentioned above, the updated step size $\mu_{s}$ of the secondary path modeling process is taken to be 0.0002. When converging to the ideal state, it can be assumed that ${\hat{S}}^{\prime }(z)$ is equivalent to $S^{\prime }(z)$ at this point. Both the active noise control program and the secondary path modeling program are written through LabVIEW software. The running and stopping of the program are controlled by an embedded controller.

## 3. Simulation Analysis

In this section, the simulation model of active noise control for a liquid-filled pipeline is established by LabVIEW software, and the control effect of line spectrum noise is simulated and analyzed. The primary pulsation in the pipeline consists of three sinusoids with frequencies of 100, 200, and 300 Hz, whose pulsation amplitude is 0.25, 0.1, and 0.05 MPa, respectively. Considering the existence of broadband background noise in the actual pipeline, a white noise signal with an amplitude of 0.01 MPa is added to the primary pulsation simulation. Program-generated coefficients based on the structural and coupling characteristics of the pipeline are used to simulate the coupled primary and secondary paths. The secondary path is estimated by an offline modeling procedure. As a comparison, the primary pulsations are actively controlled by wideband FxLMS and narrowband FxLMS algorithms, respectively. The sampling rate of the analog signal is set to 2000 Hz.

**Figure 3:**
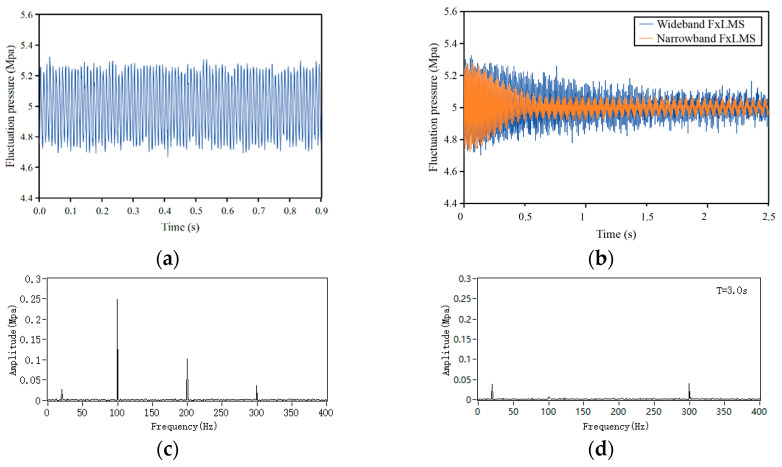
Simulation results. (**a**) Primary pulsation time-domain waveform; (**b**) comparison of wideband FxLMS and narrowband FxLMS control effects; (**c**) primary pulsation frequency-domain waveform; (**d**) residual pulsation frequency-domain waveform after narrowband FxLMS control.

The simulation results of active noise control are shown in Figure 3. A comparison of the control effects of the conventional wideband FxLMS and the notch narrowband FxLMS algorithms is illustrated in Figure 3b. The notch narrowband FxLMS algorithm converges at around 0.5 s after the active control starts. It can be observed that the control effect and convergence speed of narrowband FxLMS on line spectral noise are significantly better than that of wideband FxLMS. After iterative updating of the weight coefficients, the residual pressure pulsation tends to be stable at T = 1 s, in which the noise signals of 100 Hz and 200 Hz are almost completely suppressed. The pressure pulsation of 300 Hz is not well controlled, which may be due to the secondary path modeling mismatch.

**Figure 4:**
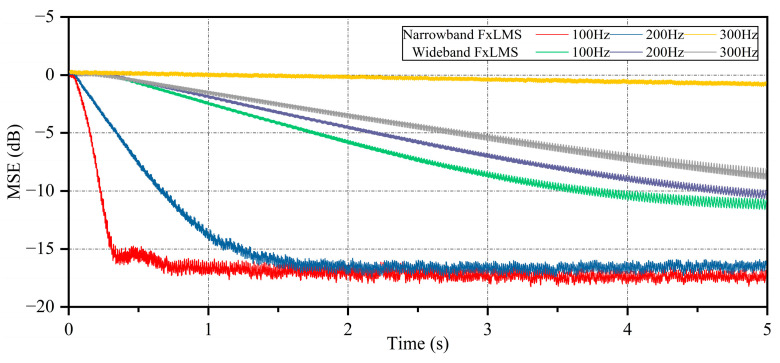
Mean square error learning curves for active noise control algorithms at different frequencies.

The mean square error (MSE) learning curves of the wideband FxLMS algorithm and the narrowband FxLMS algorithm are plotted through simulation. As shown in Figure 4, it can be observed that the narrowband FxLMS algorithm converges quickly to −15 dB at 100 Hz and 200 Hz. Compared to the wideband FxLMS algorithm, the narrowband FxLMS algorithm with notch filters updates the filter weight coefficients by the synthesized signals and thus can take a relatively larger step size for fast convergence. However, the convergence of the narrowband FxLMS is problematic at the 300 Hz position. It can be speculated that the phase error due to the secondary path modeling at this frequency is large. The simulation results show that the active control method based on coupled signal acquisition can effectively reduce most of the low-frequency line spectrum noise in the pipe.

## 4. Active Noise Control Test

**Figure 5:**
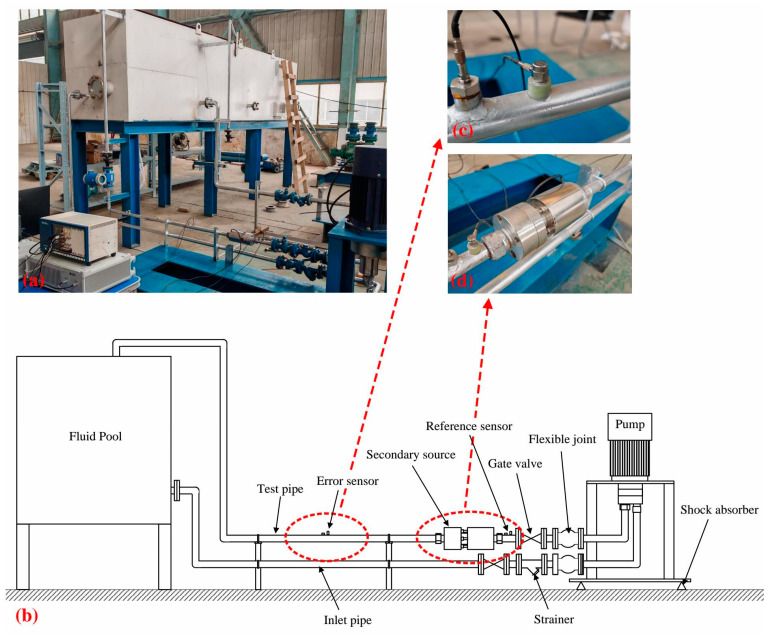
Pipeline circulation test system. (**a**) Test environment; (**b**) schematic diagram of the test system; (**c**) accelerometers for error signal acquisition and dynamic pressure sensors for noise level monitoring; (**d**) intrusive secondary source.

In this paper, the active noise control test of a liquid-filled pipeline is carried out by setting up a pipeline circulation test system. The test system mainly consists of several parts, including a high-pressure piston pump, a water reservoir, a water intake line, a test line, a data acquisition and control system, and a secondary source. The corresponding installation position of each part is shown in Figure 5b. The maximum outlet pressure of the high-pressure piston pump is 15 Mpa, the rated speed is 700~1800 rpm, and the flow rate is 15 cm^2^/rev. The motor speed of the pump unit is regulated by the power cabinet. The data acquisition and control system includes sensors, controllers, analog inputs and outputs, and power amplifiers. For the sensor part, two accelerometers (Brüel & Kjær Type 4513-B-001, sensitivity from 1 to 50 mV/ms^−2^) are used to measure the vibration acceleration of the pipe wall, and two dynamic pressure sensors are used to monitor the pressure pulsation of the liquid in the pipeline. In the test, the accelerometers are used as reference and error sensors, which are responsible for obtaining the reference and error signals to update the weight coefficients of the controller. The dynamic pressure sensors are then used to monitor the control effect of the noise in the pipeline. The analog input module (NI PXIe-4464) provides the signals collected on the individual sensors to the embedded controller (NI PXIe-8861). The embedded controller provides the output parameters of the adaptive filter to the power amplifier through an analog output module (NI PXIe-4463). The power amplifier then generates the electrical signals that control the secondary source actuation. The test pipe section is made of DN25 steel pipe with a wall thickness of 3.5 mm. When the system is in operation, the fluid is extracted from the reservoir by a high-pressure plunger pump through the water inlet pipe and returned to the reservoir after passing through the secondary source and test pipe.

During the test, the secondary path containing the coupled path was first modeled and then active noise control was performed. Active noise control studies were conducted for pulsation noise at 900 rpm, 1050 rpm, 1200 rpm, and 1350 rpm operating conditions. The adaptive notch narrowband FxLMS algorithm is used to attenuate the low-frequency line spectrum under different operating conditions. The operation of the active noise control program, data acquisition, and recording are controlled through LabVIEW software.

## 5. Results and Discussion

**Figure 6:**
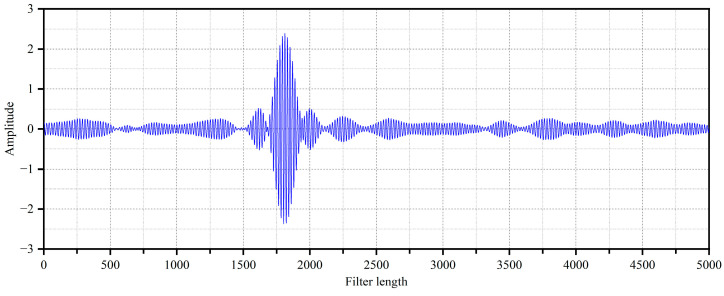
Secondary path impulse response curve.

The secondary path modeling filter is a transverse FIR filter of order 5000. After the offline modeling program was run, the modeling filter began path modeling the test pipe from the secondary source to the error sensor. The filter’s weight coefficients converged at about 20 s. The impulse response curve of the coupled secondary path obtained by the secondary path modeling program is shown in Figure 6.

**Figure 7:**
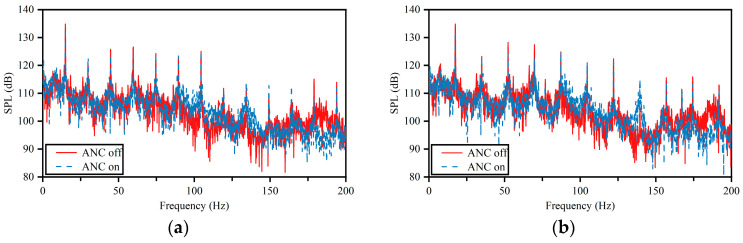

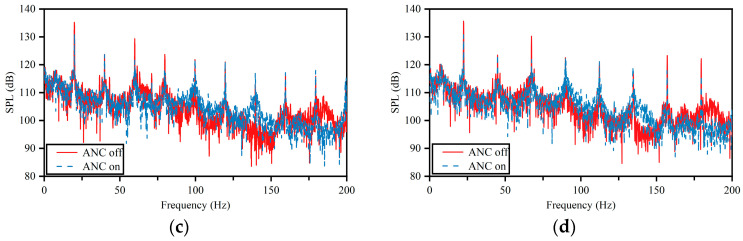
Active noise control test results. (**a**) Noise spectrum at 900 rpm; (**b**) noise spectrum at 1050 rpm; (**c**) noise spectrum at 1200 rpm; (**d**) noise spectrum at 1350 rpm.

The active noise control test was carried out through the pipeline circulation system in Figure 5. The pulsation noise at the error sensor location was measured at different operating conditions and the sound pressure levels in the pipeline were compared before and after the active noise control was turned on. The test results are shown in Figure 7, where Figure 7a–d represent the frequency domain distributions of pulsating noise when the motor speed of the pump set is 900 rpm, 1050 rpm, 1200 rpm, and 1350 rpm, respectively.

By comparing the noise spectrum before and after active noise control in Figure 7a–d, it is found that when the motor speed is 900 rpm, the control effect of active noise control is not remarkable, and only a noise reduction of approximately 1.4 dB was achieved in the frequency band ranging from 10 to 200 Hz. As the motor speed of the pump set increased to 1350 rpm, the noise reduction effect of active noise control became very significant, with a noise reduction of 4.6 dB in the frequency band ranging from 10 to 200 Hz. It can be observed that the control effect of active noise control gradually improves with the increase in speed. The noise reduction in the main noise line spectra at different speeds is shown in Table 1.

As the motor speed increases, the frequency of the main noise line spectrum changes, and its values are highly consistent with the calculations of Equation (2). There are variations in the spectral noise amplitude under different speeds, which may be due to the change in the main vibration modes of the pipeline under different pulsating excitation frequencies.

The noise reduction achieved by active noise control became gradually more significant as the motor speed increased. This may be due to the fact that at higher rotational speeds, the pressure inside the pipeline is higher and the liquid-filled state is better, resulting in stronger fluid–solid coupling vibrations in the pipeline. Thus, the data collected by the accelerometers can more accurately reflect the characteristics of the pressure pulsation in the pipe.

## 6. Conclusions

In this paper, a vibration signal-based active noise control method for liquid-filled pipelines is proposed. This method collects reference and error signals through accelerometers on the pipe wall, eliminating the need to make holes in the pipe wall and bury sensors in conventional methods, which improves the structural strength and reliability of the pipeline. The update of the filter weight coefficients is controlled by the notch narrowband FxLMS algorithm, which not only reduces the amount of arithmetic but also avoids the influence of the secondary acoustic feedback and improves the effect of the control of the line spectral noise. The control effect of active noise control at different rotational speeds was experimentally tested. With the increase in the motor speed of the pumping unit, the noise reduction on the frequency band of 10~200 Hz increased from 1.4 dB to 4.6 dB. It was found that the control effect at high rotational speeds was better than that at low rotational speeds, which may be due to the low pressure in the pipe at low rotational speeds and the weaker fluid–solid coupling. The results obtained from simulations and experiments show that effective control of the pulsation noise in the pipe can be realized by processing the acceleration signals acquired from the sensors.

Due to the introduction of coupled paths in this method, a higher order of the secondary path modeling filter is required. Modeling mismatches may occur when modeling secondary paths, affecting the effectiveness of controlling some of the line spectra. Future research will focus on improvements in secondary path modeling algorithms to reduce steady-state errors and speed up convergence in secondary path modeling.

## Figures and Tables

**Table 1:** Noise reduction in different frequency line spectra under different motor speeds.

Motor Speed/rpm	Frequency/Hz	Noise Reduction Amount/dB
900	15	1.3
	44.8	2.2
	59.7	3.4
1050	17.5	3.4
	52.3	4.1
	69.7	5
1200	19.9	4.3
	59.8	7.7
	79.7	5.9
1350	22.5	4.9
	67.3	9.4
	157	5.4

## Data Availability

The original contributions presented in this study are included in the article material, and further inquiries can be directed to the corresponding author.
